# Gamma Knife Radiosurgery With Mask Fixation Under General Anesthesia for Pediatric Patients

**DOI:** 10.7759/cureus.20905

**Published:** 2022-01-03

**Authors:** Hideo Yamaguchi

**Affiliations:** 1 Department of Medical Technology, Osaka City General Hospital, Osaka, JPN

**Keywords:** high-definition motion management system, gamma knife icon, pediatric patients, mask fixation, general anesthesia, gamma knife radiosurgery

## Abstract

Gamma knife radiosurgery (GKS) is performed on children by frame fixation of the skull under general anesthesia. With the introduction of the Gamma Knife Icon, treatment by fixing with a thermoplastic mask has become possible. In this study, we performed GKS by mask fixation under general anesthesia, measured the accuracy, and examined whether an accuracy equivalent to that of frame fixation could be guaranteed.

We included three children who underwent mask fixation under general anesthesia between September and November 2020. After the induction of general anesthesia, a patient marker was attached to the nose, and the movement of the marker before mask fixation was measured using a real-time high-definition motion management (HDMM) system. The movement of the patient marker from the start to the end of treatment after mask fixation was monitored and measured.

After the induction of general anesthesia, the movement of the patient marker was ≤0.3 mm in two cases and ≥1.0 mm in one case without mask fixation. When the mask was fixed, it was ≤0.2 mm in all three cases. It was confirmed that the marker could move even under general anesthesia without mask fixation, and it could be suppressed to a minimum after mask fixation.

With the mask fixation method devised in this study, GKS under general anesthesia for children seems to be a safe and highly accurate method.

## Introduction

A gamma knife (GK) is a device for radiosurgery dedicated to the head that uses gamma rays emitted from the radionuclide cobalt-60. When irradiating an intracranial target with high-dose gamma rays, it is essential to fix the head with a frame in order to reduce the positional error to <1 mm [1,2]. However, fixing an aluminum frame (Leksell G-frame) to the patient's skull using a titanium screw is an invasive method. For adults, this procedure is possible under local anesthesia. However, for children, general anesthesia is often required. Furthermore, since children’s skulls are thinner than those of adults, there is a risk of complications such as skull fracture and epidural hematoma [3]. Therefore, GK radiosurgery (GKS) has not been indicated for children less than two years of age.

GK Icon is equipped with cone-beam computed tomography (CBCT) and a real-time high-definition motion management (HDMM) system, enabling stereotactic radiosurgery by fixing a frameless mask. In this study, we aimed to measure the accuracy of treatment by mask fixation under general anesthesia in pediatric patients and examine its usefulness.

## Technical report

**Figure 1:**
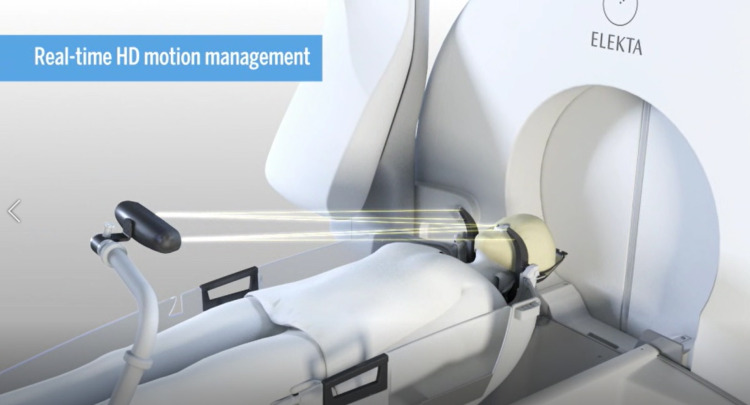
Real-time high-definition motion management system Image courtesy: Elekta Company, Stockholm, Sweden.

The HDMM system attached to the GK unit consists of an infrared camera attached to the side of the treatment table corresponding to the patient’s legs, and four infrared reference tools were fixed to the GK head support system. This system monitors the movement of the marker attached to the tip of the patient’s nose (Figure 1).

Irradiation is automatically interrupted when the movement of the marker exceeds the set threshold value (usually 1.0-1.5 mm) during treatment, and it is resumed when the marker returns to a position within the threshold value within 30 seconds of the interruption. Another CBCT must be performed if the marker does not return to a position within the threshold value within 30 seconds. Additionally, if the threshold is exceeded five times per shot, it is necessary to use another CBCT to correct the planned dose distribution.

**Figure 2:**
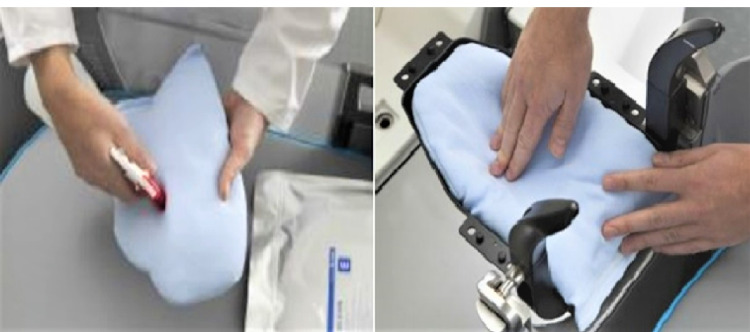
Head and neck cushion Image courtesy: Elekta company.

**Figure 3:**
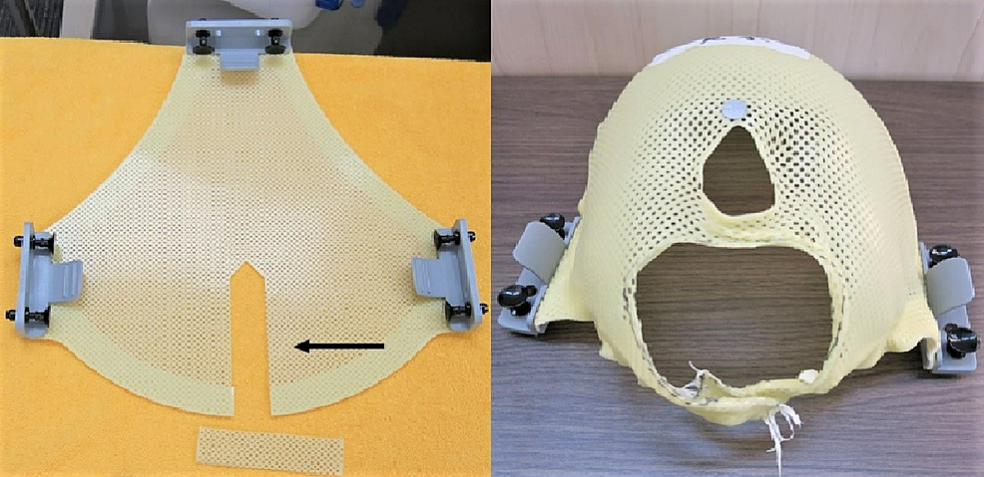
Thermoplastic mask for general anesthesia Slit is added at the mandibular area (arrow).

We used a head and neck cushion as well as a thermoplastic mask to fix the head (Figures 2 and 3).

**Figure 4:**
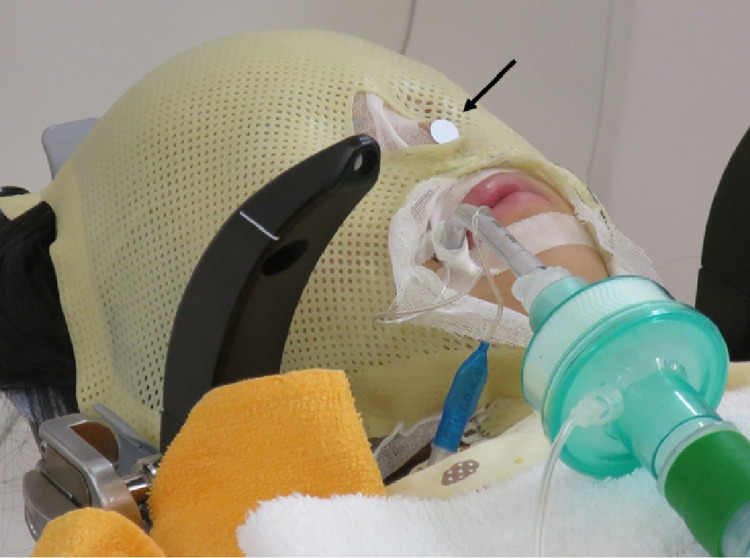
Patient marker Patient marker is placed at the tip of the patient’s nose (arrow).

The former forms and fixes the contour from the back of the head to the neck of the patient. After soaking the cushion in a small amount of water, we made it blend in and then shaped it. The solidification took approximately eight minutes (Figure 2). Thermoplastic masks should first be made with a notch to avoid an endotracheal intubation tube. We warmed it with hot water at 70 °C for about 10-30 minutes to soften it, and then formed it according to the contour of the patient's face. It took approximately three minutes to solidify (Figure 3). These operations are critical because they are related to the accuracy of the fixing. The patient marker was attached to the tip of the patient's nose (Figure 4).

**Figure 5:**
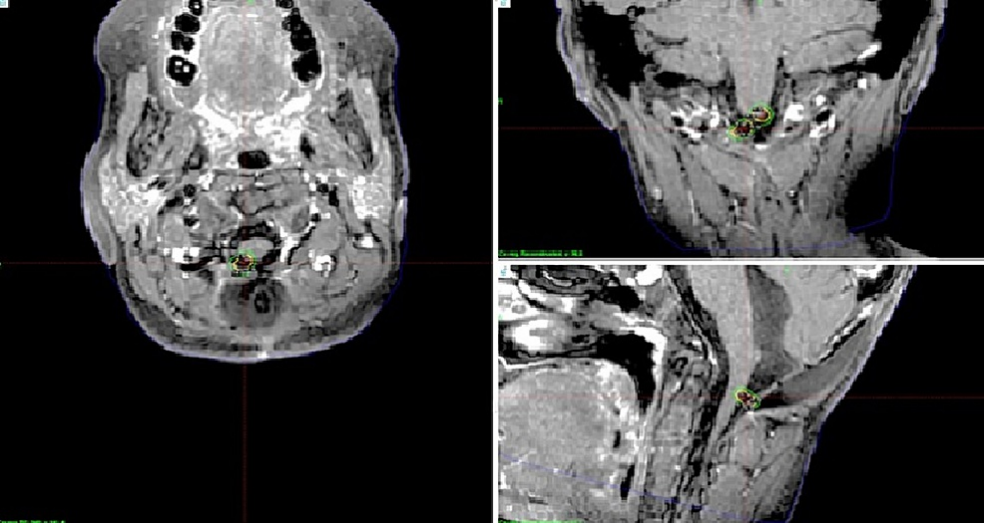
Case 1 Magnetic resonance images of a nine-year-old girl with recurrent ependymoma. Two disseminated small lesions were treated with the prescribed dose of 14 Gy at the tumor margin. Irradiation took 31.5 minutes.

**Figure 6:**
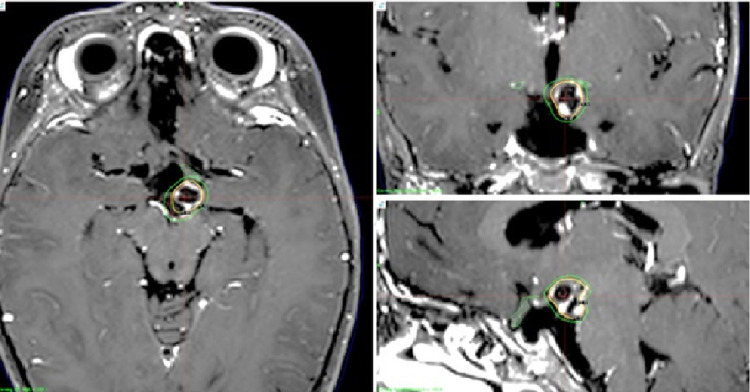
Case 2 Magnetic resonance images of a two-year-old girl with recurrent craniopharyngioma. A small left suprasellar lesion was treated with the prescribed dose of 14 Gy at the tumor margin. Irradiation took 32.8 minutes.

**Figure 7:**
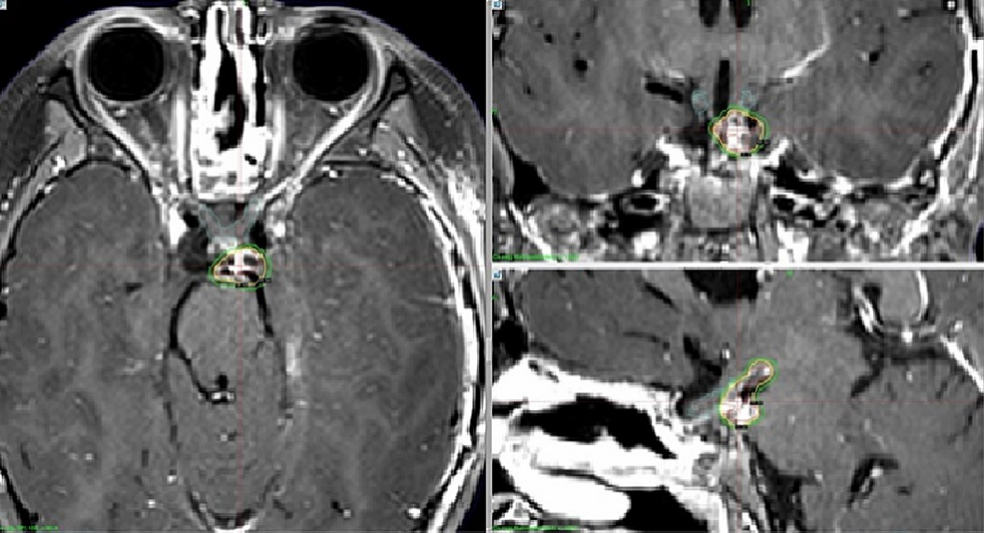
Case 3 Magnetic resonance images of a five-year-old girl with recurrent craniopharyngioma. A small suprasellar tumor was treated with the prescribed dose of 13 Gy at the tumor margin. Irradiation took 76.8 minutes.

We treated three children with mask fixation under general anesthesia between September and November 2020. Case 1 was an ependymoma in a nine-year-old girl; Case 2 was a craniopharyngioma in a two-year-old girl; and Case 3 included a five-year-old girl (Figures 5-7).

After the induction of general anesthesia, we soaked the head and neck cushion in water and placed it on the mask adapter to shape it. While paying attention to the intubation tube, we moved the patient’s head on the cushion and fixed it from the back of the head to the neck. A patient marker was attached to the tip of the nose, and the movement of the tip before mask fixation was measured using the HDMM system. After the intubation tube was taped to the cheek, gauze was placed over the face to prevent adhesion of the tape to the thermoplastic mask, following which the mask was fixed from above. When making a mask, it is especially important to firmly fix the frontal region, base of the nose, and mandible. Since children have small bodies, the height of the treatment table must be raised. However, the treatment table can be moved up and down only along the head side. Therefore, when the head side is raised, the foot side becomes relatively low, and as a result, the body tends to shift toward the foot side. To prevent this, we placed a non-slip sheet on the treatment table in advance. The movement of the patient marker from the start to the end of treatment was monitored and measured using the HDMM system.

**Figure 8:**
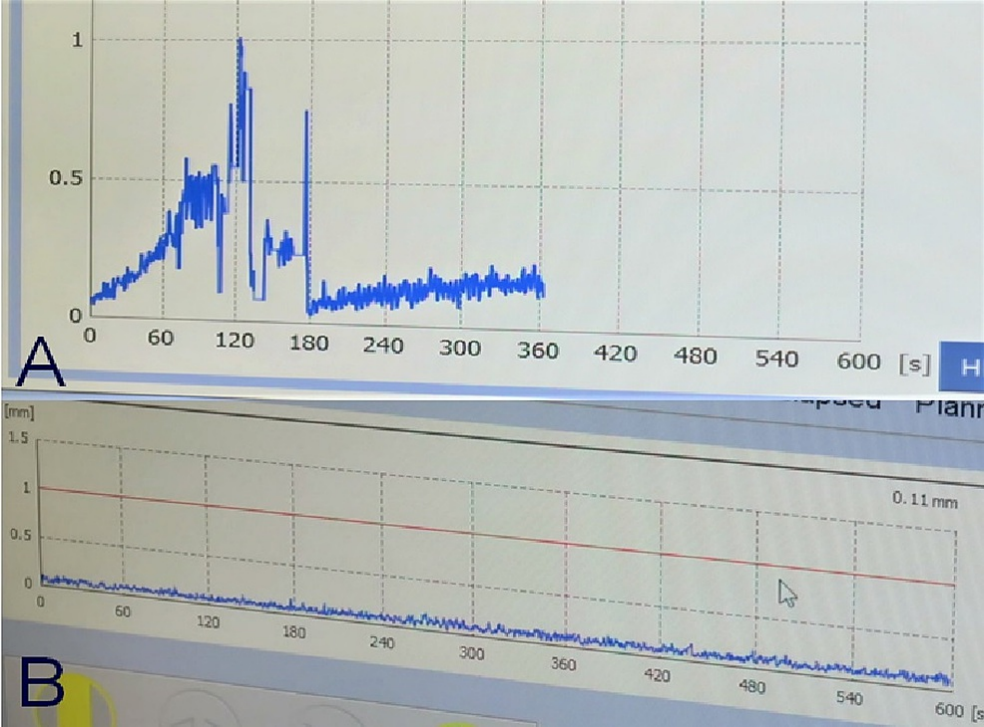
Display screen of the console during the treatment of patient 1 Movement prior to fixation by the mask was approximately 0.3 mm or less (A). After mask fixation, the movement was within 0.2 mm (B).

Case 1 was a nine-year-old girl with ependymoma who was irradiated with 14 Gy at the margin of two small disseminated lesions in the bilateral medulla oblongata with an irradiation time of 31.5 minutes. The movement of the marker before fixing the mask was ≤0.3 mm (Figure 8A). The movement after fixing the mask was maintained at ≤0.2 mm. The movement was small even before the mask was fixed, but the accuracy was higher after the mask was fixed (Figure 8B).

**Figure 9:**
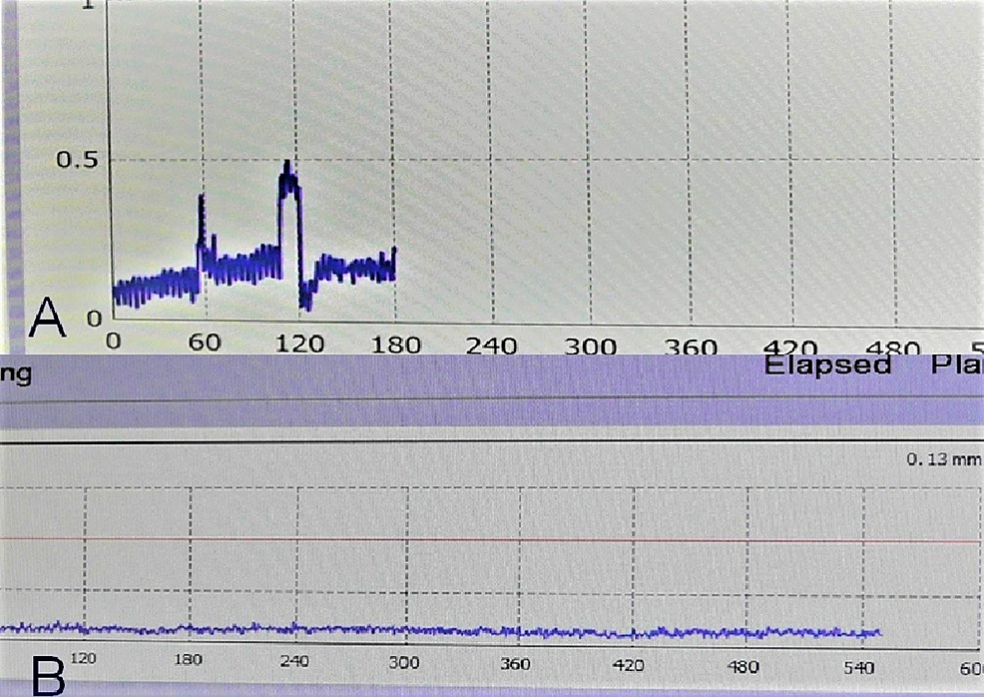
Display screen of the console during the treatment of patient 2 Movement prior to mask fixation was <0.3 mm (A). The large movement of approximately 1 mm can be attributed to the procedure for mask preparation. After mask fixation including treatment, the movement was within 0.2 mm (B).

Case 2 was a two-year-old girl with a recurrent craniopharyngioma. The left suprasellar lesion was irradiated with 14 Gy to the margin, and the irradiation time was 32.8 minutes. The movement before fixing the mask was ≤0.3 mm (Figure 9A). After fixation, the patient's movement was maintained at ≤0.2 mm (Figure 9B).

**Figure 10:**
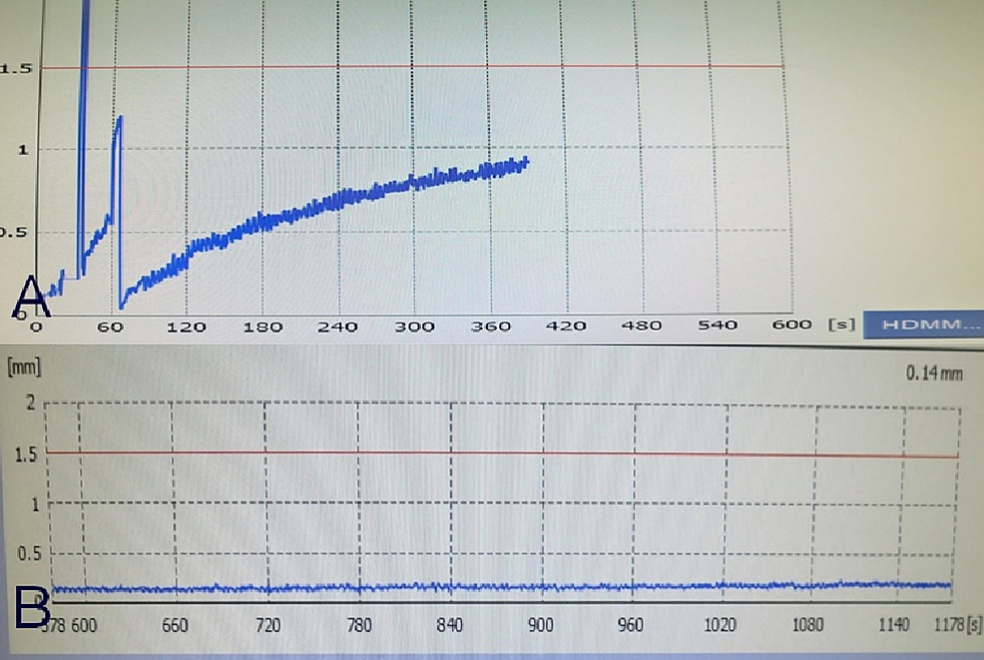
Display screen of the console during the treatment of patient 3 Movement prior to mask fixation was >1 mm (A). After mask fixation, it was <0.2 mm (B).

Case 3 was a five-year-old girl with recurrent craniopharyngioma who was irradiated with 13 Gy on the margin of a small suprasellar lesion, with an irradiation time of 76.8 minutes. The movement before fixing the mask was ≥1 mm (Figure 10A). The movement after fixing was maintained at ≤0.2 mm (Figure 10B).

It was confirmed that there was movement even under general anesthesia without mask fixation, and the movement could be suppressed after fixation.

## Discussion

Since the dose distribution of gamma rays emitted from the GK unit drops sharply from the edge of the target to the outside, the treatment is greatly affected by the position error. To minimize this error, the skull was fixed conventionally. Frame fixation was also necessary to recognize the position of the head in three-dimensional space. However, while fixing the frame has the advantage of reducing errors, it is invasive. Adults can be treated under local anesthesia, but children require general anesthesia until a certain age.

In their report, Vitali et al. reported that the incidence of depressed fractures and epidural hematoma associated with fixation of Mayfield’s head clamp for craniotomy in children was 0.65% [3]. Girhin et al. reported that the average thickness of the skull in children under 12 years old is 3.7-6.1 mm for the frontal temporal region and 3.9-5.9 mm for the occipital parietal region. Therefore, the authors stated that the safe age for using pins for Halo Vest fixation is three years or more [4]. Further attention should be paid to patients after craniotomy. As a preventive measure against fractures, it is considered effective to take a preoperative CT scan to check the thickness of the skull and to use a torque wrench for fixation.

**Figure 11:**
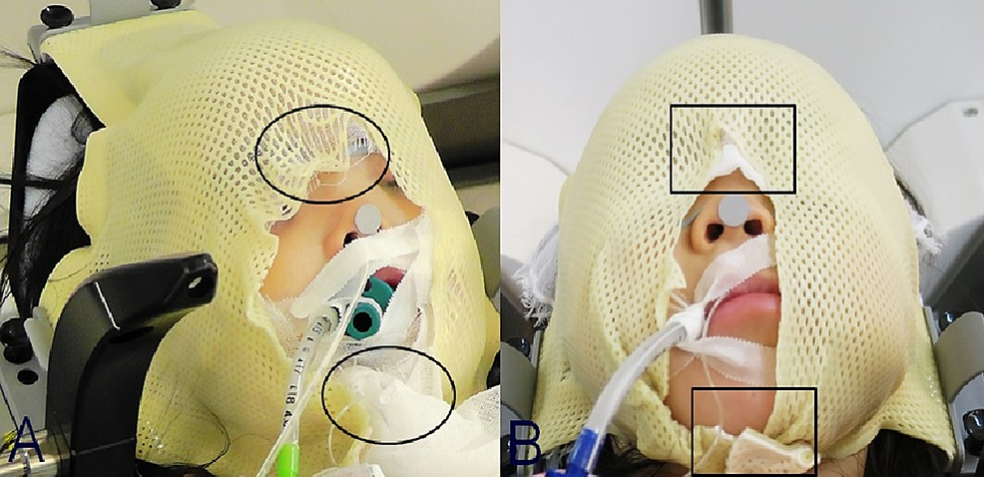
Mask fixation An example of inadequate mask fixation. The forehead, nasal root, and mandible are not fixed sufficiently (A, circle). An example of appropriate mask fixation is also shown. The forehead, nasal root, and mandible are fixed firmly (B, box).

From our results, the motion error associated with mask fixation under general anesthesia for pediatric patients was within 0.2 mm in all three patients, which is considered satisfactory. However, mask fixation is required, even under general anesthesia. We believe that fixation tends to be rough when a mask is not made in the correct manner. When the mask is being made, the patient’s mandible must be covered, and the base of the nose and forehead should be firmly fixed (Figure 11).

With the introduction of GK Icon, GKS for pediatric patients now has more options for treatment by mask fixation as well as treatment by frame fixation under general anesthesia. Since the treatment is non-invasive and safer than frame fixation, general anesthesia may not be necessary depending on the patient’s age. Although frames have been fixed under general anesthesia in children around 10 years of age, there is a possibility that treatment by mask fixation can be performed while the patient is awake. Moreover, mask fixation can be safely used in cases where there is difficulty in fixing the frame in patients <2 years of age as well as in children after craniotomy.

In the future, performing GKS with mask fixation on children will become a mainstream method. However, currently, as angiographic images are required for the treatment of cerebral arteriovenous malformations, the use of an indicator box is indispensable, and frame fixation is essential.

## Conclusions

Using the mask-fixing method we devised, GKS by mask fixation under general anesthesia for children is considered to be a safe method while maintaining high accuracy. Since there is no risk of skull depression associated with frame fixation, it is thought that treatment for children <2 years of age is possible. General anesthesia may not be necessary depending on the patient's age. Performing GKS with mask fixation on children will become mainstream.
